# Effects of fructose added to an oral glucose tolerance test on plasma glucose excursions in healthy adults

**DOI:** 10.1016/j.metop.2023.100245

**Published:** 2023-05-12

**Authors:** Amée M. Buziau, Jean L.J.M. Scheijen, Coen D.A. Stehouwer, Casper G. Schalkwijk, Martijn C.G.J. Brouwers

**Affiliations:** a Department of Internal Medicine, Division of Endocrinology and Metabolic Disease, Maastricht University Medical Center+, Maastricht, the Netherlands; b CARIM School for Cardiovascular Diseases, Maastricht University, Maastricht, the Netherlands; c Department of Internal Medicine, Division of General Internal Medicine, Laboratory for Metabolism and Vascular Medicine, Maastricht University, Maastricht, the Netherlands; d Department of Internal Medicine, Maastricht University Medical Center+, Maastricht, the Netherlands

**Keywords:** Oral glucose tolerance test, Fructose, Glucose metabolism, Blood glucose, Oral fructose, Humans

## Abstract

**Background and objective:**

Previous experimental studies have shown that fructose interacts with glucose metabolism by increasing hepatic glucose uptake. However, human studies investigating the effects of small (‘catalytic’) amounts of fructose, added to an oral glucose load, on plasma glucose levels remain inconclusive. The aim of this study, therefore, was to repeat and extend these previous studies by examining the plasma glucose response during a 75 g oral glucose tolerance test (OGTT) with the addition of different doses of fructose.

**Methods:**

Healthy adults (n = 13) received an OGTT without addition of fructose and OGTTs with addition of different doses of fructose (1, 2, 5, 7.5 and 15 g) in a random order, on six separate occasions. Plasma glucose levels were measured every 15 min for 120 min during the study**.**

**Findings:**

The plasma glucose incremental area under the curve (iAUC) of the OGTT without addition of fructose was not significantly different from any OGTT with fructose (p ≥ 0.2 for all fructose doses). Similar results were observed when these data were clustered with data from a similar, previous study (pooled mean difference: 10.6; 95%CI: 45.0; 23.8 for plasma glucose iAUC of the OGTT without addition of fructose versus an OGTT with 5 g fructose; fixed-effect meta-analysis, n = 38). Of interest, serum fructose increased from 4.8 μmol/L (interquartile range: 4.1–5.9) at baseline to 5.3 μmol/L (interquartile range: 4.8–7.5) at T = 60 min during an OGTT *without* addition of fructose (p = 0.002).

**Conclusion:**

Low doses of fructose added to an OGTT do not affect plasma glucose levels in healthy adults. The role of endogenous fructose production, as a potential explanation of these null-findings, deserves further investigation.

## Highlights

• Fructose doses added to an oral glucose load do not affect plasma glucose levels.
• There is a non-linear relationship between fructose dose and serum fructose levels.
• Serum fructose levels increased after an oral glucose load *without* fructose.

## 1 Introduction

The rise in intake of added sugars has been associated with the current epidemic of obesity, type 2 diabetes mellitus (T2D), dyslipidemia, and cardiovascular disease [[1], [2], [3], [4]]. Recent studies have shown that fructose, more than glucose, is disadvantageous for cardiometabolic health [[5], [6], [7]], which may be explained by the fact that fructose is preferentially metabolized in the liver resulting in, among others, intrahepatic lipid accumulation and hepatic insulin resistance [[1], [2], [3], [4]].

Of interest, findings of previous experimental studies suggest that fructose also interacts with glucose metabolism by increasing hepatic glucose uptake (Supplemental Fig. 1) [[8], [9], [10], [11], [12]]. First, *in vitro* studies have shown that small (‘catalytic’) amounts of fructose dissociate glucokinase from glucokinase regulatory protein, a liver-specific protein, which results in more free, cytosolic glucokinase that facilitates the conversion of glucose to glucose-6-phosphate [8]. Second, experimental studies in dogs and humans have shown that these ‘catalytic’ amounts of fructose increase hepatic glucose uptake [9,10]. Third, Moore et al. elegantly showed that adding 7.5 g of fructose to a 75 g oral glucose tolerance test (OGTT) reduced plasma glucose excursions, most likely due to increased hepatic glucose uptake, in both healthy adults and individuals with T2D [11,12]. However, it is currently not known at what threshold fructose interacts with glucose *in vivo*. In fact, a more recent study [13], could not replicate the findings reported by Moore et al. [11].

Therefore, the aim of the present study was to repeat and extend the original study by Moore et al. by studying the plasma glucose response during an OGTT with and without the addition of different doses of fructose (ranging from 1 g to 15 g).

## 2 Research design and methods

### 2.1 Participants and experimental design

Thirteen healthy adults were studied on six separate occasions with at least a four-day interval (Supplemental Fig. 2 + 3).

Participants visited the research ward in the morning after an overnight fast (10:00 p.m.) and remained fasted prior to the OGTT. All participants completed a health questionnaire regarding, among others, medical history and medication use. Height was determined using a stadiometer. Weight was measured by using electronic scales. BMI was calculated as weight in kilograms divided by height in meters squared. Waist circumference was determined using a measuring tape at the level of the umbilicus, measured while participants were in a standing position.

A 20-gauge intravenous cannula was inserted on the dorsal side of the hand for blood sampling at baseline and after ingestion of the carbohydrate solution every 15 min for a total of 120 min during the study. The hand was placed in a thermostatically controlled warmed box throughout the study to obtain arterialized venous blood samples [11,12].

Participants were instructed to ingest 82.5 g dextrose monohydrate (= 75 g glucose; fructose content ≤ 0.15%; Tereos, Aalst, Belgium), with or without addition of different fructose doses (Nutricia, Scholten, the Netherlands), dissolved in 250 mL water over the course of 5 min. Participants were blinded and randomly received in total six different carbohydrate solutions dissolved in 250 mL water during each study visit, including: 1) OGTT without addition of fructose, 2) OGTT with 1 g fructose, 3) OGTT with 2 g fructose, 4) OGTT with 5 g fructose, 5) OGTT with 7.5 g fructose, and 6) OGTT with 15 g fructose (Supplemental Fig. 2 + 3).

The study was carried out according to the Declaration of Helsinki [14] and approved by the medial ethical committee of Maastricht University Medical Center+. All participants provided written informed consent.

### 2.2 Laboratory measurements

Plasma glucose levels were determined every 15 min by using the YSI2300 STAT Plus Glucose Lactate Analyser (YSI, Yellow Springs, OH). Serum fructose concentrations were measured with a recently developed and validated Ultra Performance Liquid Chromatography–tandem Mass Spectrometry method [15]. Serum lipids were measured by an enzymatic colorimetric assay (Cobas 8000 instrument, Roche Diagnostics, Mannheim, Germany). Low-density lipoprotein cholesterol was calculated using the Friedewald formula.

### 2.3 Statistical analysis

Data are presented as median with interquartile range or as frequencies in case of continuous and categorical variables, respectively (unless stated differently).

The trapezoidal rule was used for the calculation of the incremental area under the curve (iAUC). Wilcoxon signed-rank tests (unless stated differently) were used to compare between the plasma glucose iAUC during an OGTT without addition of fructose and plasma glucose iAUC during the OGTTs with different doses of fructose.

Statistical analyses were performed with the use of the Statistical Package for Social Sciences (Version 25.0; IBM, Chicago, IL) and the ‘R’ statistical software (R Developmental Core Team) using the metaphor package [16]. Results were considered statistically significant at p < 0.05.

## 3 Results

### 3.1 Population characteristics

Participants were predominantly male and, on average, not overweight (Table 1). None of them were diagnosed with T2D.

**Table 1:** Baseline characteristics.

	Overall population (n = 13)
Sex (M/F)	11/2
Age (y)	24 (21–45)
Smoker (yes/no)	0/13
BMI (kg/m^2^)	23.5 (22.0–25.8)
Waist circumference (cm)	84.0 (76.6–89.5)
Systolic blood pressure (mmHg)	121 (111–133)
Diastolic blood pressure (mmHg)	70 (68–78)
Total cholesterol (mmol/L)	3.7 (3.5–4.4)
HDL-cholesterol (mmol/L)	1.4 (1.2–1.8)
LDL-cholesterol (mmol/L)	2.0 (1.6–2.6)
Triglycerides (mmol/L)	0.7 (0.5–0.9)
Glucose (mmol/L)	5.2 (5.0–5.4)

Categorical data presented as frequencies and continuous data as median (IQR).

HDL: high-density lipoprotein; LDL: low-density lipoprotein.

### 3.2 Plasma glucose response

**Fig. 1:**
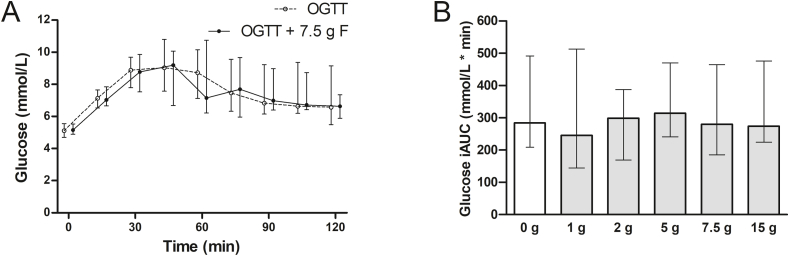
Glucose response during an OGTT without addition of fructose and OGTTs with different doses fructose (n = 13). Panel A: Plasma glucose concentrations during a 75 g oral glucose tolerance test (OGTT) with addition of 0 g and 7.5 g fructose (F); panel B: Plasma glucose incremental area under the curve (iAUC) during a 75 g OGTT with addition of 0 g, 1 g, 2 g, 5 g, 7.5 g, and 15 g fructose. Data are presented as median (IQR).

Plasma glucose levels were not statistically significantly lower at any time point during an OGTT with 7.5 g fructose when compared to an OGTT without addition of fructose (Fig. 1A), nor during an OGTT with 1 g, 2 g, 5 g, 15 g fructose when compared to an OGTT without addition of fructose (Supplemental Fig. 4). Moreover, the plasma glucose iAUC was not significantly different between an OGTT without addition of fructose and OGTTs with addition of different doses of fructose (p ≥ 0.2 for all fructose doses versus OGTT without addition of fructose; Fig. 1B).

### 3.3 Sensitivity analyses

To gain more insight into these null-findings, we performed additional analyses.

First, we clustered our data (n = 13) with the individual data (n = 25) that were kindly provided by Braunstein et al. [13]. Unfortunately, individual data from the experiments by Moore et al. were no longer available (Moore; personal communication) [11,12]. A fixed-effect meta-analysis of the available data (n = 38) did not show a significantly lower plasma glucose iAUC after 5 g fructose added to an OGTT when compared to an OGTT without addition of fructose (pooled mean difference: 10.6; 95% CI: 45.0; 23.8; Supplemental Fig. 5).

**Fig. 2:**
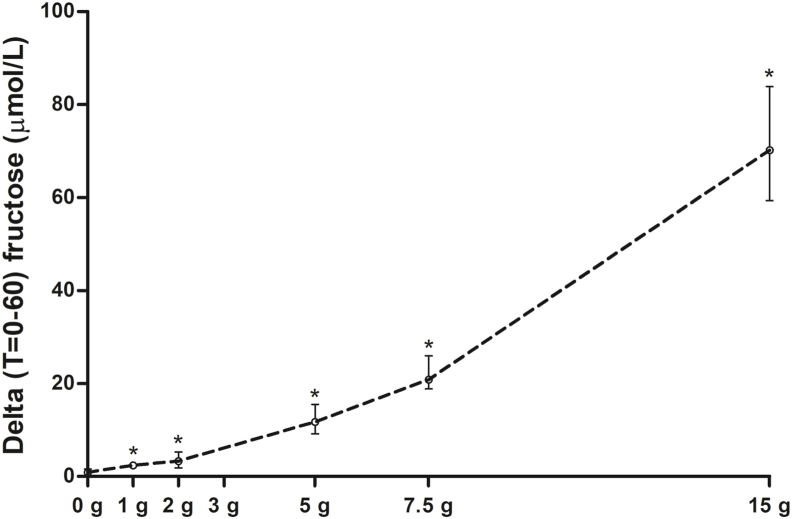
Fructose response during OGTTs with addition of different doses of fructose (n = 13). Delta serum fructose (from baseline to T = 60 min) during a 75 g oral glucose tolerance test (OGTT) with addition of 0 g, 1 g, 2 g, 5 g, 7.5 g, and 15 g fructose. Data are presented as median (IQR). *, p < 0.05 versus OGTT without addition of fructose. Analysed with Wilcoxon signed-rank tests.

Second, other experimental studies have shown that fructose is also metabolized in the intestines, thereby preventing fructose spill over to the liver [[17], [18], [19]]. To gain insight into the degree of spill over (from intestines *and* liver), we measured the serum fructose response (T = 0 and T = 60 min) during the OGTTs with the different fructose doses. An exponential relationship was observed between the different doses of fructose added to an OGTT and the serum fructose response (Fig. 2).

**Fig. 3:**
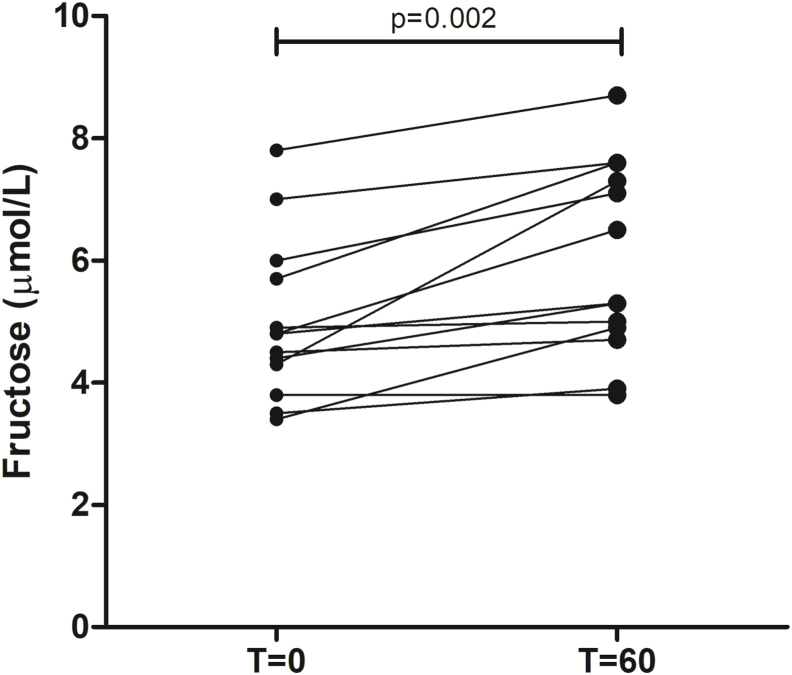
Fructose response during an OGTT *without* addition of fructose (n = 13). Serum fructose concentrations at T = 0 and T = 60 min during a 75 g oral glucose tolerance test (OGTT) *without* addition of fructose. Data are presented as median (IQR). Analysed with Wilcoxon signed-rank tests.

Third, previous experimental studies have shown that high intracellular glucose concentrations stimulate endogenous fructose production via the polyol pathway (Supplemental Fig. 1) [20]. We observed a small, statistically significant increase in serum fructose (from baseline to T = 60 min) during the OGTT *without* addition of fructose (p = 0.002; Fig. 3).

## 4 Discussion

In the present study, we did not find an effect of oral fructose on the plasma glucose response during an OGTT in healthy adults.

Our findings are in contrast with those reported by Moore et al. who showed that adding 7.5 g of fructose to an OGTT reduced plasma glucose excursions in both healthy adults (n = 11) and individuals with T2D (n = 5) [11,12]. However, our findings are in agreement with a recent study from Braunstein et al. who also could not replicate Moore's findings by adding 5 g or 10 g fructose to an OGTT in healthy adults (n = 25) [13]. Although there were some subtle differences in the study design of these four OGTT studies [[11], [12], [13]], including blinding, the total number of the OGTTs, wash-out period between the OGTTs, and the utility of a thermostatically controlled heated box, it is unlikely that these could account for the observed discrepancy.

Of interest, Braunstein et al. found that self-reported ethnicity was a significant effect modifier for the effect of fructose on the plasma glucose iAUC (p = 0.04), i.e. the plasma glucose iAUC was higher during an OGTT with fructose in three individuals who self-reported their ethnic category as ‘other’ [13]. Similarly, Moore et al. reported a higher plasma glucose iAUC with addition of 7.5 g fructose to an OGTT in two Asian males [11]. However, the present study included only Caucasians and, therefore, ethnicity cannot explain the higher plasma glucose iAUC during some OGTTs with addition of fructose in our study.

Finally, statistical power could be an issue. The reproducibility (and accuracy) of a glucose response during an OGTT is in general poor and dependent on numerous variables [21]. Therefore, in a sensitivity analysis, we clustered our data with the data reported by Braunstein et al. [13], which did not materially alter the results.

In order to gain more biological insight into these null-findings and explain the discrepancy with other *in vivo* studies (in dogs and humans) showing that fructose favours hepatic glucose uptake [[8], [9], [10]], we performed additional sensitivity analyses. First, since animal studies have shown that intestinal fructose metabolism scavenges fructose away from the liver (and peripheral circulation) [[17], [18], [19]], we studied the serum fructose response during an OGTT with addition of different doses of fructose. We observed a non-linear relationship between the fructose dose and the serum fructose response, indeed suggesting that at lower doses less fructose escapes the intestinal (and hepatic) fructose metabolism. On the other hand, we did detect a statistically significant increase in serum fructose after fructose doses even as little as 1 g and 2 g. This suggests that at least some fructose passes the small intestine and reaches the liver favouring dissociation of glucokinase from glucokinase regulatory protein.

Second, in another sensitivity analysis, we studied the serum fructose response during an OGTT *without* addition of fructose. We found a small, statistically significant increase in serum fructose levels. Although the oral glucose might have contained trace amounts of fructose (maximum 0.15% [according to the manufacturer] * 82.5 g = 0.1 g), we believe that this amount is too low to explain the observed increase in serum fructose. It is, therefore, more likely that the increase in serum fructose during an OGTT *without* addition of fructose is explained by endogenous fructose production via the polyol pathway [20]. Indeed, Francey et al. performed a stable isotope study and showed that endogenous fructose production was increased 60 min after oral intake of 30 g of glucose [22]. Hence, it possible that the amount of endogenously produced fructose is already sufficient to maximally dissociate glucokinase from glucokinase regulatory protein, explaining why the addition of exogenous fructose to an OGTT did not affect plasma glucose excursions (Supplemental Fig. 1). This would imply that repeating the OGTT with lower doses of glucose – below the threshold of endogenous fructose production – might yield different results.

## 5 Conclusions

We did not find an interaction between oral glucose and low doses of fructose on plasma glucose excursions in healthy adults. The potential role of endogenous fructose production deserves further investigation.

## Funding sources

This work was supported by the 10.13039/501100003092Dutch Diabetes Research Foundation (Grant 2017.82.004; MCGJ Brouwers).

## CRediT authorship contribution statement

**Amée M. Buziau:** Conceptualization, Methodology, Resources, Formal analysis, Writing – original draft, Writing – review & editing. **Jean L.J.M. Scheijen:** Resources, Methodology, Writing – review & editing. **Coen D.A. Stehouwer:** Resources, Methodology, Writing – review & editing. **Casper G. Schalkwijk:** Resources, Methodology. **Martijn C.G.J. Brouwers:** Conceptualization, Methodology, Resources, Funding acquisition, Formal analysis, Writing – original draft, Writing – review & editing.

## Declaration of competing interest

The authors declare that they have no known competing financial interests or personal relationships that could have appeared to influence the work reported in this paper.
