# Supplementary material for: Effects of fructose added to an oral glucose tolerance test on plasma glucose excursions in healthy adults
Source: Metabol Open. 2023 May 12;18:100245. doi: 10.1016/j.metop.2023.100245 (PMC10209703; doi:10.1016/j.metop.2023.100245)
Supplement: Multimedia component 1 [file mmc1.docx]

**Supplemental material**

**
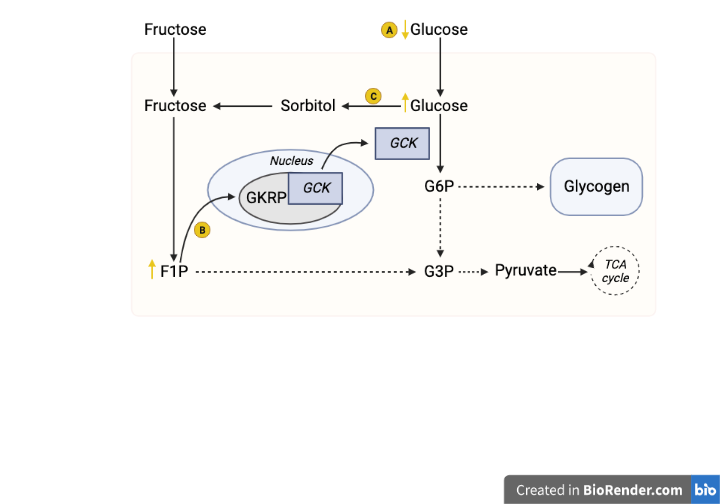
**

**Supplemental Figure 1. Overview of fructose and glucose metabolism in the liver.**

(**A**) Findings of previous experimental studies have shown that fructose interacts with glucose metabolism by increasing hepatic glucose uptake, i.e. fructose 1-phosphate (F1P) dissociates glucokinase (GCK) from glucokinase regulatory protein (GKRP), which results in more free, cytosolic GCK that facilitates the conversion of glucose to glucose-6-phosphate (G6P). (**B**) Increased cytosolic GCK favours hepatic glucose uptake, resulting in lower plasma glucose levels; **(C)** Previous studies have shown that high intracellular glucose concentrations stimulate endogenous fructose production via the polyol pathway.

Dashed arrow indicates multiple intermediate enzymatic steps that have not been visualized for simplicity purposes.

**
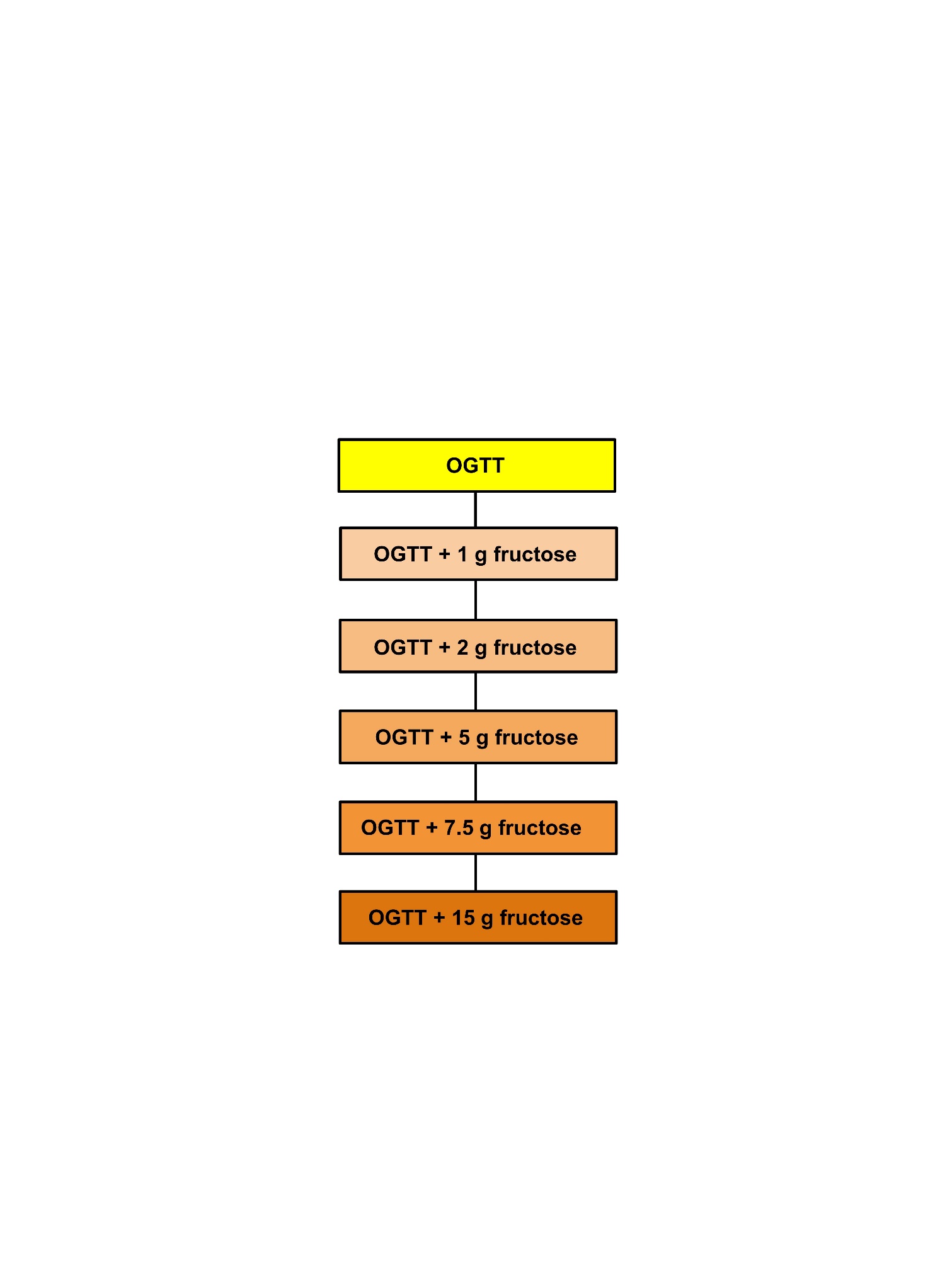
**

**Supplemental Figure 2. OGTT experiments in healthy adults (n=13).**

A 75 g oral glucose tolerance test (OGTT) without addition of fructose and OGTTs with addition of 1 g, 2 g, 5 g, 7.5 g, and 15 g fructose.

**
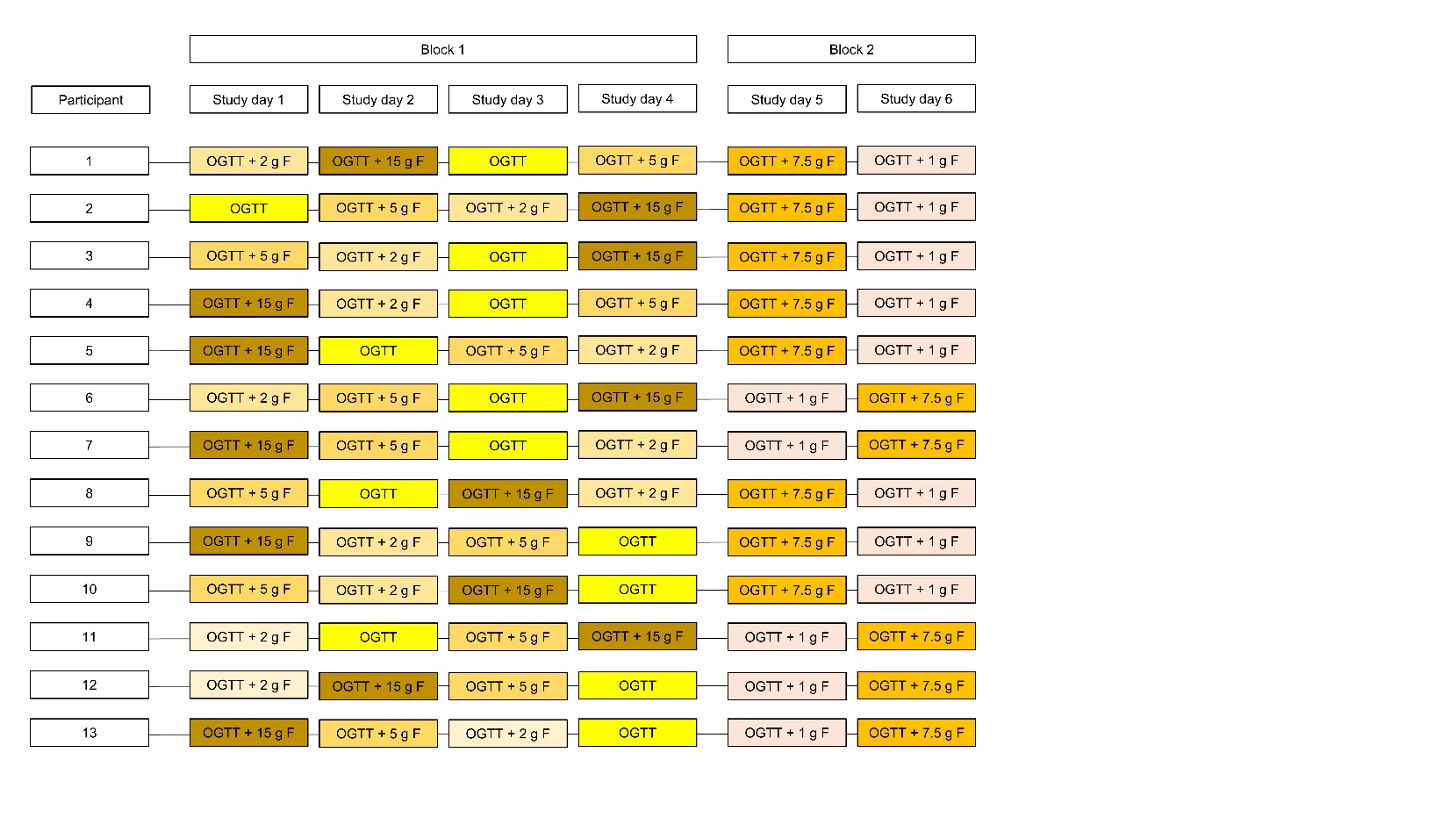
**

**Supplemental Figure 3. Block randomization of the OGTT experiments in healthy adults (n=13).**

Block 1 includes a 75 g oral glucose tolerance test (OGTT) without addition of fructose (F) and three OGTTs with addition of the three most distinctive doses of fructose (i.e. 2 g, 5 g, and 15 g).

**Supplemental Figure 4. Glucose response during an OGTT without addition of fructose and OGTTs with different doses fructose (n=13).**


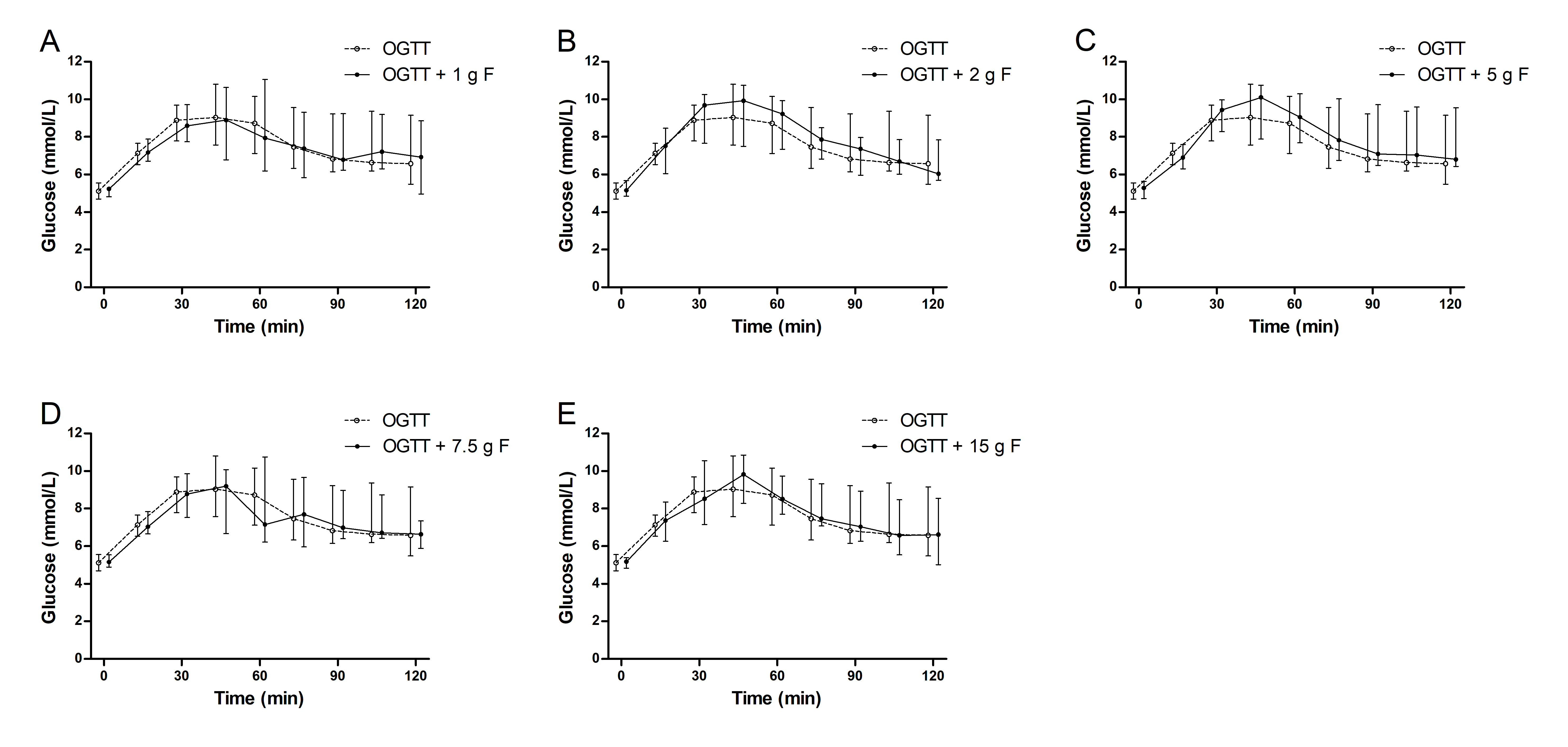


Plasma glucose concentrations during a 75 g oral glucose tolerance test (OGTT) with addition of 0 g (reference) and 1 g (**A**), 2 g (**B**), 5 g (**C**), 7.5 g (**D**), and 15 g (**E**) fructose (F). Data are presented as median (IQR).

**
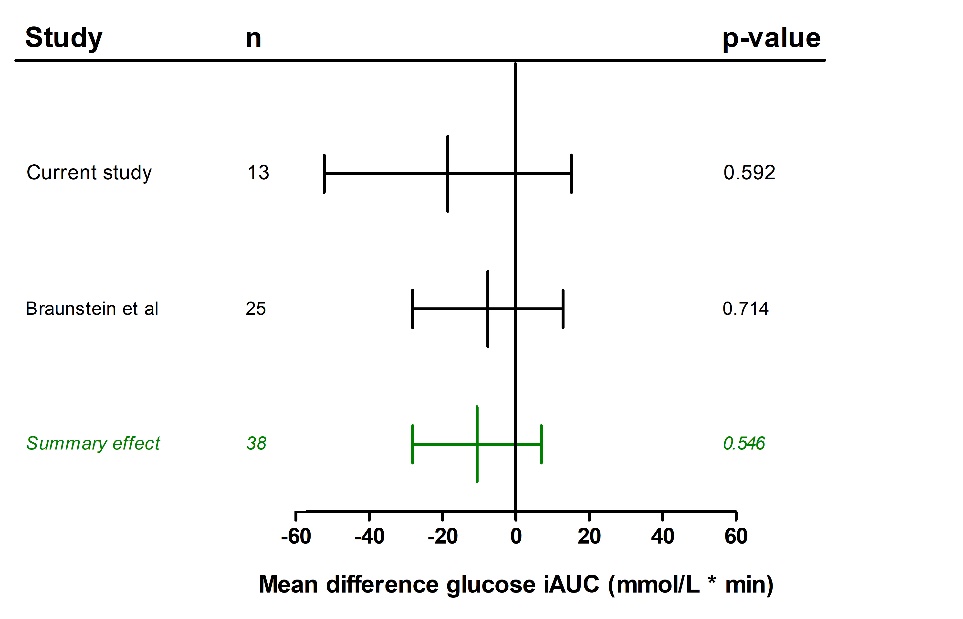
**

**Supplemental Figure 5. Mean difference in glucose response between an OGTT without addition of fructose and an OGTT with 5 g fructose.**

Mean difference in plasma glucose incremental area under the curve (iAUC) during a 75 g oral glucose tolerance test (OGTT) without addition of fructose versus an OGTT with 5 g fructose. Data are presented as mean difference ± SEM.

Analysed with paired T-tests for individual data and a fixed-effect meta-analysis for summary effect.
